# The prevalence, risk factors and outcomes of anaemia in South African pregnant women: a systematic review and meta-analysis

**DOI:** 10.1186/s13643-022-01884-w

**Published:** 2022-01-25

**Authors:** Vinogrin Dorsamy, Chauntelle Bagwandeen, Jagidesa Moodley

**Affiliations:** 1 Laboratory Medicine and Medical Sciences, College of Health Sciences, University of KwaZulu-Natal, Durban, South Africa; 2 Department of Public Health Medicine, School of Nursing and Public Health, University of KwaZulu-Natal, Durban, South Africa; 3 Women’s Health and HIV Research Group, School of Clinical Medicine, University of KwaZulu-Natal, Durban, South Africa

**Keywords:** Pregnant, Anaemia, Maternal, Haemoglobin, South Africa

## Abstract

**Background**

Anaemia is associated with maternal and perinatal morbidity and mortality. The pooled prevalence of anaemia in the South African (SA) pregnant population was ascertained by systematically reviewing available literature. Severity, risk factors (HIV, tuberculosis, race, province, year of study), maternal morbidity and mortality (hypertensive disorders of pregnancy), birth outcomes (including low birth weight) and supplementation during pregnancy were also described.

**Methods**

Eligible studies reported on haemoglobin concentration or prevalence of anaemia in a SA pregnant population and were available in full text. Case-control and estimation studies were excluded with no restriction on the date of publication. PubMed, CINAHL, EMBASE, EBSCO, Ovid maternity and infant care databases, Cochrane Database of Systematic Reviews, Web of Science and SCOPUS were searched, using the keywords ‘anaemia’, ’haemoglobin’, ‘pregnancy’, ‘South Africa’. Risk of bias was conducted using the Hoy tool and the Doi plot and LFK ratio. Overall study quality was assessed using the GRADE tool. Due to heterogeneity amongst studies subgroup analyses were performed (random effects and quality effects model) using MetaXL addon tool for Microsoft Excel.

**Results**

The initial search yielded 7010 articles and 26 were selected for inclusion. Twenty studies were cross-sectional, three were longitudinal and one a randomised control trial. Studies ranged in publication year from 1969 to 2020. The pooled prevalence of anaemia in pregnant women in SA was determined to be 31% (95% CI, 23–40%). Hypertensive disorders of pregnancy and low birth weight were associated with anaemia. While iron deficiency was reported as the main cause, other risk factors included HIV and other infections.

**Discussion**

Limitations

There was limited data reporting on prevalence of anaemia and direct maternal and foetal outcomes. Heterogeneity amongst studies was not explained by subgroup analysis. Majority of cross-sectional study designs reduced the ability to infer causality.

Interpretation

While the prevalence of anaemia remains high and of concern, risk factors are varied. Iron deficiency is still common but the presence of comorbidities also contributes to anaemia and should not be ignored. More longitudinal research into associations between anaemia and birth outcomes is needed due to a lack of available evidence.

**Systematic review registration**

PROSPERO 2020: CRD42020157191.

**Supplementary Information:**

The online version contains supplementary material available at 10.1186/s13643-022-01884-w.

## Background

Anaemia is a multifactorial disorder, defined as a reduced oxygen-carrying capacity of the blood or a decrease in red blood cells or haemoglobin. Its aetiology is affected by socioeconomic disparity, environmental threats and genetic propensity [1]. A condition of global public health concern [2], it affects around a quarter of the world’s population, especially those in low- and middle-income countries (LMICs). Iron deficiency anaemia is the commonest type. Vulnerable population groups such as pre-school children and women of reproductive age are most affected [3]. Pregnant women who need to accommodate the haemodynamic demands of pregnancy may be at higher risk for anaemia, which is increased by exposure to synergistic inflammatory and infective conditions. This can lead to complications for both mother and baby, such as spontaneous abortion, preterm delivery, low birth weight and/or developmental delay in infants, with increased mortality in severe cases [4, 5]. If not controlled, future pregnancies are worse affected [6, 7].

The World Health Organisation (WHO) recommends universal iron prophylaxis to reduce the impact of anaemia during pregnancy [8]. It is important to understand both the prevalence and causative factors of anaemia in pregnancy as iron supplementation may only treat iron deficiency anaemia. Without establishing the cause of anaemia, particularly in areas where parasites or communicable diseases are endemic, such measures are not only ineffective but possibly dangerous [9–11]. Supplying iron to pregnant women who may be iron replete may risk a transitory iron overload which has also been found to be associated with adverse obstetric outcomes, as the foetus restricts hepcidin production to cater for its iron need [5, 12, 13]. While indiscriminate iron supplementation may be of benefit in a population where malnutrition is a problem [14], it is imperative to establish the nature of the anaemia where differential aetiologies may be apparent [13, 15].

By systematically reviewing the literature of studies conducted in this population, we may better understand the type and associated outcomes of anaemia in this population and modify the strategy to combat it, rather than relying on routine treatment protocols which are geared at addressing nutritional insufficiency. As far as we are aware, a systematic review of the prevalence of anaemia has not been conducted in the country. The main aim of this study was to establish the pooled prevalence of anaemia in SA pregnant women. The specific objectives and outcomes were to describe prevalence, severity, risk factors (HIV, tuberculosis, race group, year in which study published), maternal morbidity and mortality (hypertensive disorders of pregnancy (HDP)), birth outcomes (including low birth weight) and supplementation during pregnancy associated with anaemia.

## Methodology

This study was performed according to the published protocol [16] registered in the International Prospective Register of Systematic Reviews (PROSPERO 2020: CRD42020157191). The Preferred Reporting Items for Systematic Reviews and Meta-analysis 2020 (PRISMA2022) guidelines [17] were used to inform the structure of this review.

### Search strategy

Search terms used and their synonyms were identified using the Medical Subject Headings (MeSH). The uniterms and Boolean operators in English used in the search strategies were (anemia OR anaemia OR haemoglobin OR hemoglobin OR haematocrit OR hematocrit) AND (Pregnancy OR Pregnant women OR Gravidity OR Maternal exposure OR Mother OR Pregnant OR Gravid OR Obstetric OR Antenatal OR Antepartum OR Gestation) AND (South Africa OR Southern Africa or South African or Sub-Saharan Africa). The search strategy included all electronic databases as listed in the protocol complemented by a manual search of the reference list of primary articles (Additional file 2).

### Study selection

The eligibility of retrieved studies was assessed using the criteria listed in the protocol [16]. Full-text articles focussing on anaemia (< 11mg/dL of Hb) in pregnancy without restrictions on study design, setting or date of publication were included. Studies were excluded if they did not report on haemoglobin concentrations or any other accepted method of anaemia determination, were not available in full text, were case-control studies which did not report baseline prevalence or estimation studies which did not determine anaemia prevalence as a parameter within the study itself. We contacted the authors of three studies [18–20] that sampled the same population but reported on different outcome measures to determine the severity of anaemia in the overall sample. We included the population sample size from only one of these studies in our calculation of pooled prevalence.

### Data extraction and quality assessment

An abstract screening tool [16], using Google Forms was developed, piloted and distributed to the two reviewers. Abstract screening, followed by full article screening was conducted manually, including those articles for which an abstract was not available. Screening, data extraction and quality appraisal were conducted independently by two reviewers (VD and CB), and disagreement was resolved by independent assessment by a third reviewer (JM). The screening results were reported using a PRISMA flowchart [17].

### Risk of bias and quality assessment

The quality and risk of bias of selected studies were performed by both reviewers using a modification of the Hoy tool [21] (Additional file 3) which is a risk of bias tool similar to the Cochrane and GRADE tools, advocated for prevalence studies and [22, 23]. Briefly, the original tool consists of 10 binary items (low risk/high risk) addressing four domains of bias plus a summary risk of bias. Items 1 to 4 assess the external validity of the study (selection and nonresponse bias domains), items 5 to 9 assess the internal validity (measurement bias domain) and item 10 assesses bias related to the analysis [21]. We modified the tool to include an assessment of sample size with any study reporting prevalence of anaemia in under 200 pregnant women as high risk and excluded item 9 (‘Was the length of the shortest prevalence period for the parameter of interest appropriate?’) as our focus was anaemia in pregnant women with a finite term (Additional file 3). Studies were scored as low quality (1–3), moderate (4–6) and high quality (7–9). This overall quality score was converted to a fraction (/10) and then used in subgroup analyses as discussed elsewhere [24]. A test of interrater reliability using Cohen’s Kappa was performed with a cut point of 0.80 (indicating substantial agreement) and consensus was reached by both reviewers. Overall study quality was established using the Grading of Recommendations Assessment, Development and Evaluation (GRADE) tool [25].

### Data synthesis and analysis

All data abstracted was translated to a Microsoft Excel® (Microsoft Corporation, 2019) spreadsheet and reported prevalence data and means for outcomes of interest were calculated and analysed using the MetaXL addon [26] in Microsoft Excel® for generation of summary tables, forest plots of proportions and subgroup analysis. Heterogeneity amongst studies was checked using *I*^2^ to determine the heterogeneity of the studies. A value of over 50% was indicative of greater heterogeneity amongst included studies. In order to address the heterogeneity of the pooled prevalence, we performed subgroup analysis and multicategory inverse variance analysis using the IVHet function and the quality effects function on MetaXL [24]. Where it became evident that there was a duplication of study populations, only one study was used in the calculation of pooled prevalence to limit the threat to the validity of results [27]. We used the Doi plot and LFK ratio to visualise publication bias and asymmetry respectively [28]. A sensitivity analysis for overall pooled prevalence of anaemia was conducted in order to assess the impact of individual studies on the pooled prevalence.

## Results

### Deviation from protocol

As possible risk factors and variation in geographic, temporal and socio-economic status became evident, we deviated from the protocol to include these factors in the sub-group analysis in an attempt to explain the heterogeneity due to these differences. We included subgroup analyses of the year of study as some studies were conducted prior to the country transitioning to democracy (1994) when there was disparate access to healthcare amongst race groups. For this reason, subgroup analysis by race was also conducted rather than for genetic propensity. As some studies were conducted before the advent of the HIV pandemic, and HIV is associated with anaemia, it was important to understand the temporal variation before and after the advent of antiretroviral treatment. We used the MetaXL [24] software to calculate pooled prevalence as it provided an opportunity to factor in the study quality into the heterogeneity model, thereby increasing the precision and a suitable alternative to the random effects model for this meta-analysis [24]. A sensitivity analysis of all included studies used in the pooled prevalence calculation was also conducted to evaluate the impact removal of individual studies had on the overall pooled prevalence. The software also provided the use of the Doi plot and LFK ratio that was used instead of the funnel plot to address publication bias [28]. The plot is included in Additional file 4.

### Study selection

**Fig. 1:**
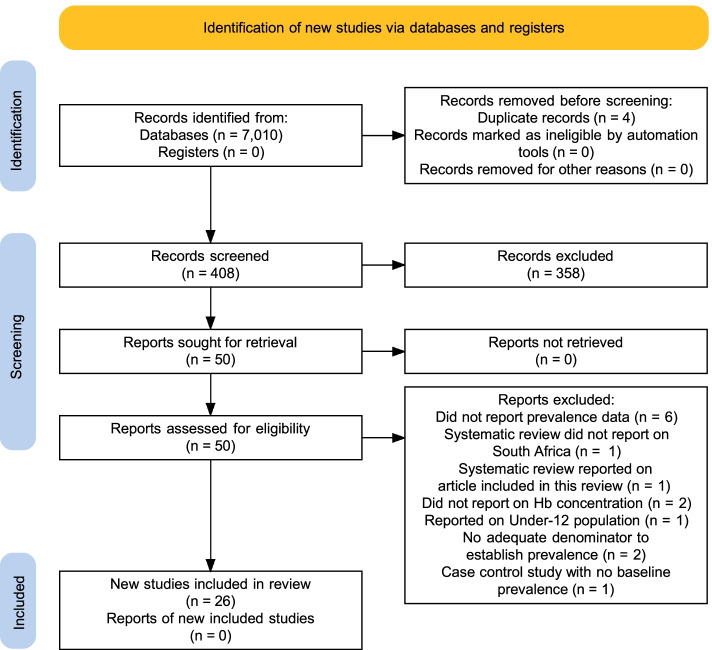
PRISMA flow diagram of the present review. Adopted from PRISMA 2020 statement [17]

A total of 7010 articles were elicited on the initial search, and 553 were selected. After removing duplicates and closely similar articles, a total of 408 remained. A total of 50 full-text articles were screened for eligibility, and 26 were selected for inclusion in this study (Fig. 1).

### Characteristics of studies

A final selection of 26 studies (Characteristics of Studies, Additional file 5) that reported on the prevalence of anaemia in pregnancy in SA were included in this meta-analysis [5, 18–20, 29–51]. Of the studies selected, 20 were cross-sectional, three studies had a longitudinal study design and one was a randomised control trial (RCT). The majority of the studies were conducted in KwaZulu-Natal province (13 studies) while in the provinces of Gauteng, Western Cape, Limpopo and the Free State there were 7, 3, 2 and 1 studies conducted respectively.

The studies ranged in publication year from 1969 to 2020. Most studies used haemoglobin (Hb) concentration alone (15 studies) as a marker of the disorder, while 10 studies used multiple methods including iron studies or a combination of Hb with ferritin and mean cell volume (MCV) to establish anaemia. One study reported a combination of Hb and B12 and one study retrospectively reviewed medical chart data where the method of determination was unclear. Iron deficiency was reported as either the cause, or most likely cause, in the majority of the studies (*n* = 17) while 9 studies did not report a cause. The total number of participants was 59235, and the mean age of participants was 24.28 years (from the 16 studies that reported on age). Only 8 studies reported on the severity of the anaemia.

### Prevalence of anaemia

**Fig. 2:**
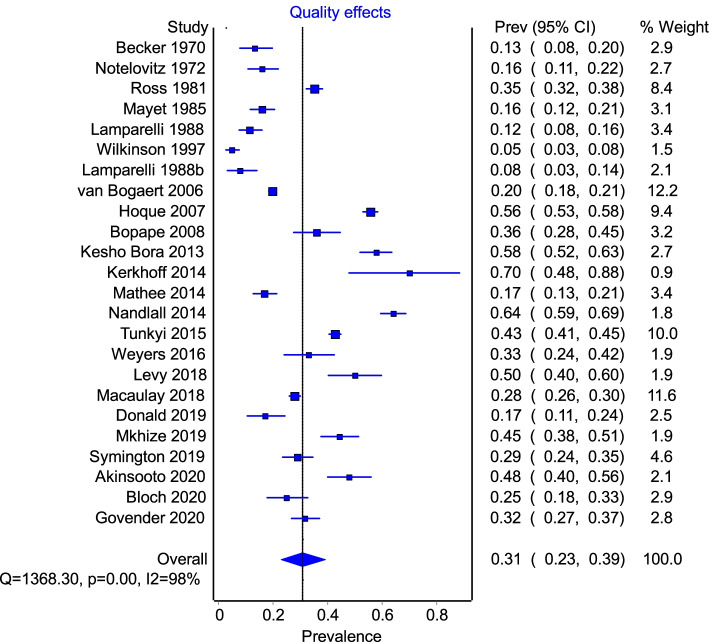
Forest plot listing proportions and the overall pooled proportion as well as *I*^2^ and Cochrane’s *Q* indicative of complete heterogeneity of studies

The pooled prevalence of anaemia derived from the selection of studies was 31% (95% CI, 23–39%) based on 24 studies depicted in the forest plot (Fig. 2) using a quality effects model [52]. The *I*^2^ was 98% suggestive of significant heterogeneity, reflective of differences in sampled populations in SA. Subgroup analysis by province (Additional file 1) revealed that KZN has a prevalence of 38% (95%CI, 24–53%), Gauteng 22% (95%CI, 16–29%) and other provinces were grouped together due to fewer studies conducted in these provinces (prevalence 41% (95%CI, 24–58%).

A Doi plot with an LFK ratio was generated to assess the risk of bias and revealed minor asymmetry (Additional file 4). A sensitivity analysis was conducted to evaluate the impact a single study had on the prevalence data (Table 1) which showed little variation apart from the removal of the van Bogaert study [48] which increased the prevalence by 2%.

**Table 1:** Summary and sensitivity analysis of prevalence across selected studies with column values of prevalence and low (LCI) and high (HCI) confidence intervals showing proportions. The value of the pooled prevalence change is depicted in the second column (‘Pooled prevalence proportion’) if the study to the left (‘Included study’) is removed from the calculation

Included study	Pooled prevalence proportion	LCI 95%	HCI 95%	Cochrane’s *Q*	*P*	*I*^2^	*I*^2^ LCI 95%	*I*^2^ HCI 95%
Akinsooto 2020 [30]	0.33	0.26	0.40	1526.19	0.00	98.43	98.14	98.67
Becker 1970 [32]	0.34	0.26	0.41	1507.74	0.00	98.41	98.12	98.66
Bloch 2020 [31]	0.33	0.26	0.41	1533.65	0.00	98.44	98.15	98.68
Bopape 2008 [33]	0.33	0.26	0.40	1538.45	0.00	98.44	98.16	98.68
Donald 2019 [34]	0.33	0.26	0.41	1521.18	0.00	98.42	98.13	98.67
Govender 2018 [35]	0.33	0.26	0.41	1538.07	0.00	98.44	98.15	98.68
Hoque 2007 [36]	0.31	0.24	0.38	1284.27	0.00	98.13	97.77	98.44
Kerkhoff 2014 [37]	0.33	0.26	0.40	1528.12	0.00	98.43	98.14	98.67
Kesho Bora 2013 [47]	0.32	0.25	0.40	1471.21	0.00	98.37	98.07	98.62
Lamparelli 1988 [38]	0.34	0.27	0.41	1471.28	0.00	98.37	98.07	98.62
Lamparelli 1988b [39, 53]	0.34	0.26	0.41	1495.27	0.00	98.39	98.10	98.64
Levy 2018 [40]	0.33	0.26	0.40	1527.69	0.00	98.43	98.14	98.67
Macaulay 2018 [41]	0.34	0.26	0.42	1505.33	0.00	98.41	98.11	98.65
Mathee 2014 [42]	0.33	0.26	0.41	1490.16	0.00	98.39	98.09	98.64
Mayet 1985 [43, 54]	0.33	0.26	0.41	1492.08	0.00	98.39	98.09	98.64
Mkhize 2019 [44]	0.33	0.26	0.40	1529.13	0.00	98.43	98.14	98.67
Nandlal 2014 [51]	0.33	0.26	0.40	1380.84	0.00	98.26	97.93	98.54
Notelovitz 1972 [45]	0.33	0.26	0.41	1508.80	0.00	98.41	98.12	98.66
Ross 1981 [46]	0.33	0.25	0.41	1537.91	0.00	98.44	98.15	98.68
Symington 2019 [5]	0.33	0.26	0.41	1535.92	0.00	98.44	98.15	98.68
Tunkyi 2015 [18]	0.32	0.25	0.40	1464.87	0.00	98.36	98.06	98.62
Tunkyi 2017 [20]	0.32	0.25	0.40	1464.87	0.00	98.36	98.06	98.62
Tunkyi 2018 [19]	0.32	0.25	0.40	1464.87	0.00	98.36	98.06	98.62
van Bogaert 2006 [48]	0.35	0.28	0.42	1122.85	0.00	97.86	97.43	98.23
Weyers 2016 [49]	0.33	0.26	0.40	1538.68	0.00	98.44	98.16	98.68
Wilkinson 1997 [50]	0.33	0.27	0.41	1349.66	0.00	98.22	97.88	98.51

### Severity of anaemia

**Fig. 3:**
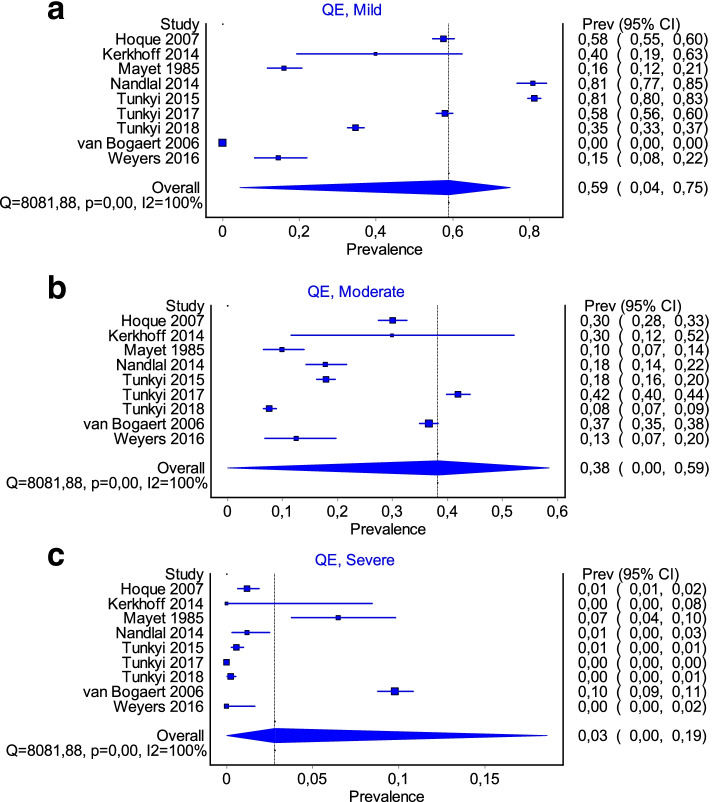
**a**-**c** Forest plots showing pooled prevalence according to the severity of anaemia grouped into **a** mild (Hb 9.0–11.0 g/dL), **b** moderate (Hb 7.0–8.9 g/dL) and **c** severe (Hb < 7.0 g/dL)

Severity of anaemia was reported in 12 studies. As depicted in the forest plots (Fig. 3a–c) the majority of the participants had a pooled prevalence of mild anaemia (59%). Moderate to severe anaemia, as reported in the different studies, was 38% and 2% respectively. heterogeneity was high (*I*^2^ 100%) even when separating for categories of severity.

### Subgroup analysis

In order to investigate the heterogeneity, sub-group analyses using study-level parameters (year of study, race, study quality, HIV) were conducted using a quality effects model. In terms of year of publication, studies were separated into three groups: pre-1994, post-1994 as that signified a change of regime (‘apartheid’ to democratic South Africa) and allowed access to all healthcare facilities by any race group, and year 2002 as this was when treatment for prevention of mother to child transmission (PMTCT) was implemented.

**Fig. 4:**
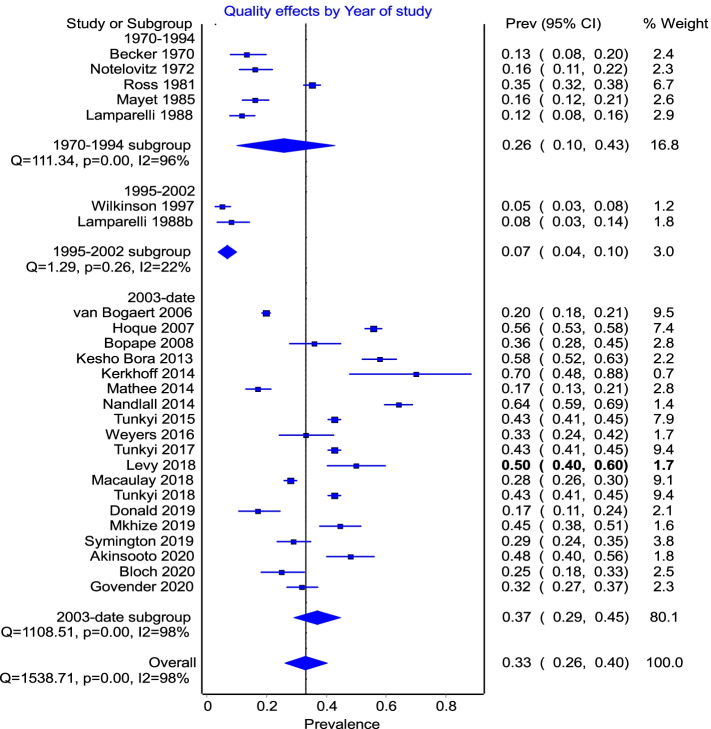
Subgroup analysis by year of study with 3 groups: pre-1994, post-1994 (abolishment of apartheid) and post-2002 (PMTCT was introduced)

The subgroup analysis (Fig. 4) showed heterogeneity in all groups but a reduction in the 2 studies in the 1995–2002 group.

**Fig. 5:**
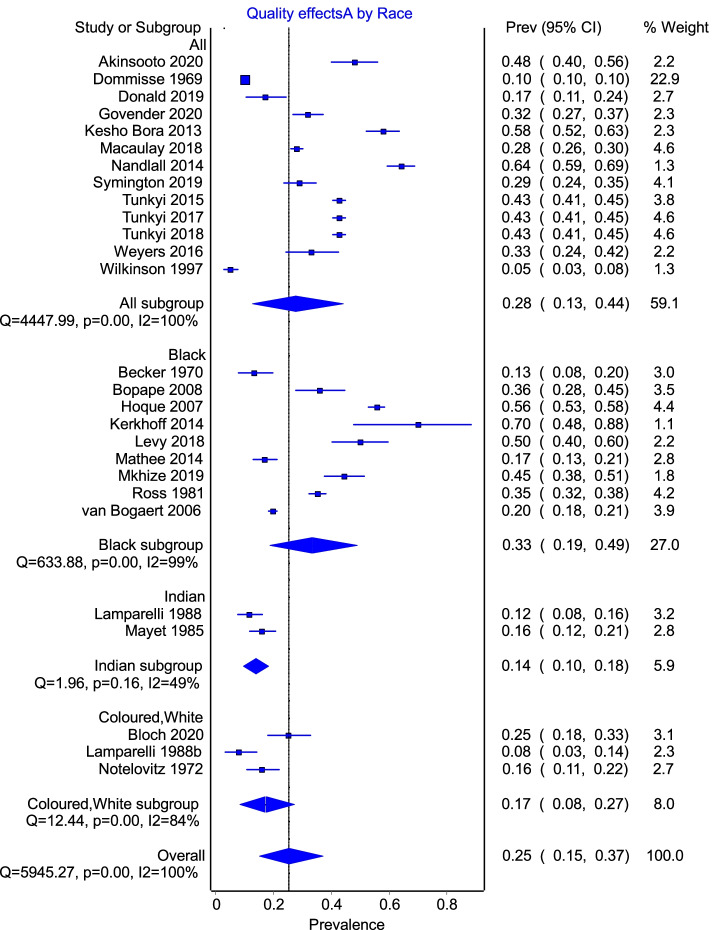
Subgroup analysis by race with 4 subgroups. Where race was not specified within studies, all races were assumed to be included within that study

As depicted in Fig. 5, a subgroup analysis of race difference between studies showed heterogeneity within subgroups apart from the Indian subgroup represented by two studies.

**Fig. 6:**
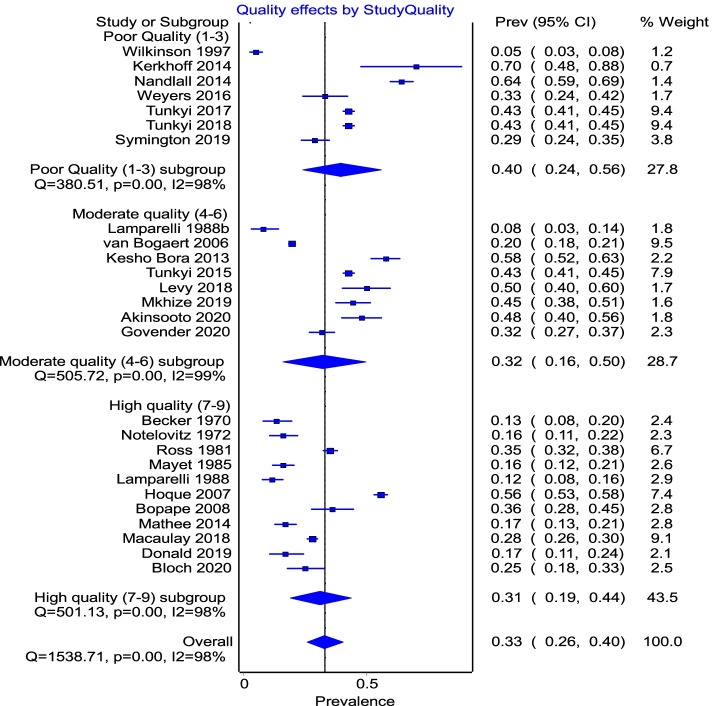
Subgroup analysis by study quality separated into low quality (1–3), moderate (4–6) and high quality (7–9) according to the Hoy tool [21] used to assess study quality

There was still significant heterogeneity amongst studies even when separated by study quality into low, moderate and high quality, and with each group showing a similar heterogeneity (*I*^2^ = 98%) within subgroups as that of the pooled result (Fig. 6). However, the poor quality group showed a higher prevalence of anaemia 40% 95%CI (24–56%) compared to the overall pooled prevalence estimate of 33% 95%CI (26–40%).

**Fig. 7:**
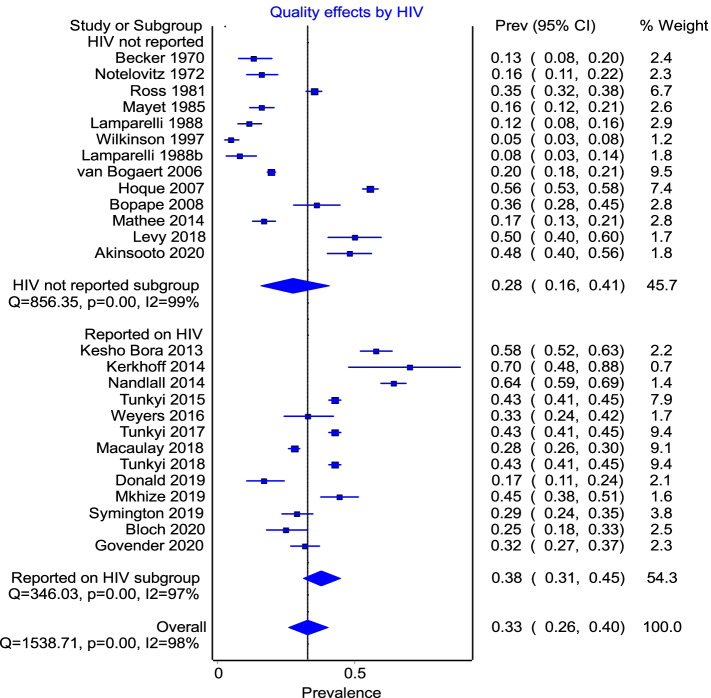
Subgroup analysis by whether studies reported on HIV as an outcome or co-factor in anaemia

A visual inspection of the overall pooled prevalence showed that anaemia was significantly higher in studies that reported on HIV as a parameter within the study. However, the forest plot (Fig. 7) showed that studies that reported on HIV did not contribute to the heterogeneity.

Eleven of the studies looked at the prevalence of HIV as a comorbidity. However, 3 of these were conducted in a cohort of positive women. Only one study looked at other common comorbidities, namely *Mycobacterium tuberculosis* (TB) [37], although others alluded to the importance of infective causes of anaemia [15, 37, 43]. In a total of 485 HIV-positive patients, Kerkhoff [51] reported that the prevalence of anaemia and TB was 55% and 16.7%, respectively, and that the prevalence of anaemia was 70% in the 20 pregnant women in the study. The prevalence of TB in this group was not reported at this level. Nandlal [51] reported that anaemic women presented more commonly with vaginal discharge or infection and urinary tract infections.

### Anaemia and birth outcomes

**Fig. 8:**
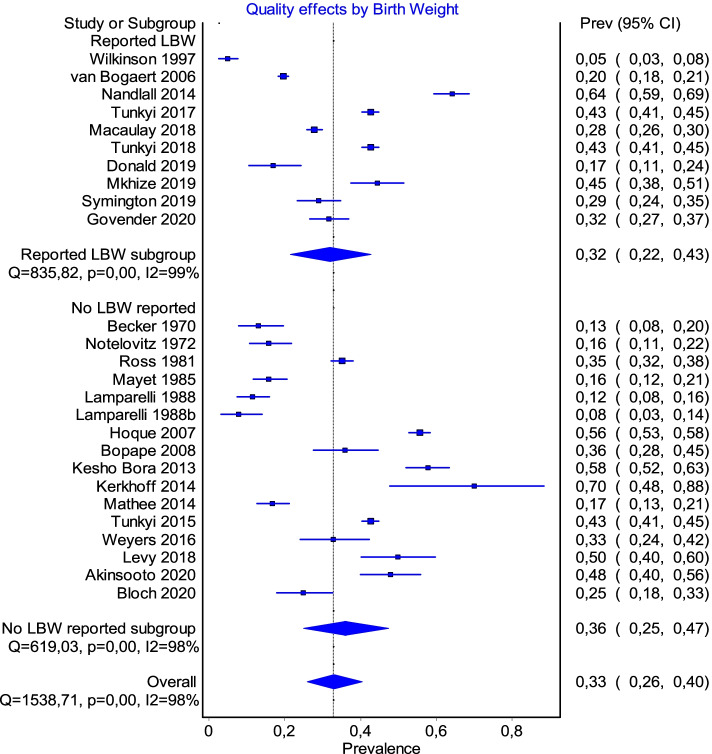
Subgroup analysis by whether studies reported on birth weight as an outcome or co-factor in anaemia

Nine studies reported on birth outcomes (Fig. 8). As there was a variety of birth outcomes discussed in the respective studies, it was not possible to further interrogate the data and individual study conclusions are thus summarised in Table 2. Tunkyi and Moodley [19] demonstrated a statistically significant association between anaemic mothers and an increase in low birth weight infants or preterm labour. In the others, relationships were either not observed, not significant, in an HIV population, or associated with late booking. However, Symington et al. [5] showed an inverse relationship between anaemic mothers and birthweight. The potential for long-term effects on delayed cognitive development of children, particularly boys born to anaemic mothers was reported by Donald [34].

**Table 2:** Maternal anaemia and birth outcomes

Author, year	Description of birth outcomes
Donald 2019 [34]	Decrease in cognitive development in children, especially boys in presence of maternal anaemia.
Govender 2018 [35]	LBW and low Apgar scores reported associated with late booking of ANC. No association determined for anaemia and birth outcome parameters.
Mkhize 2019 [44]	LBW associated with the 34-week group only.
Nandlal 2014 [51]	No difference in birth weight between groups. Infants born to mothers who presented with anaemia in pregnancy were twice more likely to be anaemic.
Nojilana 2007 [72]	IDA associated with 37% perinatal mortality and mild mental disability.
Symington 2019 [5]	Inverse relationship between birthweight and maternal anaemia.
Tunkyi 2017 [20]	Increase in placenta abruptio in patients on ARV’s.
Tunkyi 2018 [19]	LBW, preterm delivery associated with anaemia.
van Bogaert 2006 [48]	Anaemia present at early booking may predispose to caesarean section.

### Anaemia and hypertensive disorders of pregnancy

Although four studies reported on HDP, none showed a statistically significant relationship between anaemic mothers and the development of this condition (Table 3).

**Table 3:** Anaemia associated with hypertensive disorders of pregnancy

Author, year	*N* cases	Which HDP	Comments HDP
Macaulay 2018 [41]	505	Just hypertension per se	Overall prevalence 3.4%, no significant difference between diabetics and non-diabetics
Mkhize 2019 [44]	89	Pre-eclampsia	4% in > 34 weeks and 4% in < 34 weeks HIV uninfected
Nandlal 2014 [51]	262	Pre-eclampsia	5.5% in anaemia 5.4% in no anaemia RR 0.98 (0.42–2.28) *p* = 1.00
Tunkyi 2017 [20]	854	PIH	Significant difference in HIV-infected women on treatment greater than a year
Tunkyi 2018 [19]	854	PIH	Not significant

### Anaemia and supplementation

As summarised in Table 4, eleven of the studies reported on either type of supplementation provided, or the effects thereof. Iron and folate were the most common supplements, with one study also providing calcium as prophylaxis against HDP. The Kesho Bora Study Group [47] noted that some improvement in anaemia may have been due to routine supplementation, as did Mkhize et al. [39]. In general, however, there appeared to be little effect on the overall iron status of the population, despite supplementation, while Lamparelli [44] noted an exacerbation despite supplementation as the pregnancy progressed. Non-adherence to supplementation and poor records of supplementation being provided and or ingested were also observed.

**Table 4:** Anaemia and supplementation

Author, year	Supplements	Key findings
Becker 1970 [32]	Iron and folate	No difference observed in untreated subjects, and those treated with iron, and iron and folate
Bopape 2008 [33]	Iron and folate	No difference in iron, serum ferritin and B_12_ while folate higher in supplemented groups
Dommisse 1969 [55]	Oral iron (Gradumet) and injection (Imferon)	Participants responded better to injection than oral in subset of patients with severe anaemia with poor response to folate
Kesho Bora 2013 [47]	Iron, folate and multivitamins	Routine supplementation presumed to have contributed to rise in HB
Lamparelli 1988 [38, 39, 53]	Indicated iron and folate supplementation during pregnancy in affluent population	Iron stores still depleted as pregnancy progressed
Mkhize 2019 [44]	Pregamel for all and ferrous sulphate for anaemia	20.8% non-adherence overall but adherence was higher in HIV+ group. Improvement noted with adherence to haematinics
Ross 1981 [46]	Haematinics mentioned in a subgroup for longitudinal study	No difference in dietary supplementation and ferritin levels or Vit C was found between groups
Symington 2019 [5]	Ferrous sulphate, calcium and folate	100% compliance and additional supplementation in some
Tunkyi 2017 [20]	Iron and folate 200 μg	
van Bogaert 2006 [48]	Haematinics	Only 112 records indicated iron supplementation

## Discussion

This study is a comprehensive evaluation of the available literature that assessed the prevalence, risk factors and outcomes associated with anaemia in pregnant women in SA. All efforts were made to find and analyse the available data in terms of these parameters, so as to provide a unique perspective on this issue, with the aim of informing policy makers, and improve on the present basic antenatal package of care.

### Outcome: pooled prevalence of anaemia in South African pregnant women

The overall result of the meta-analysis of 26 studies gave a pooled prevalence of anaemia of 31% (95% CI, 23–39%), with an *I*^2^ of 98% indicative of the extreme heterogeneity amongst the studies. While the pooled prevalence reported in this study is in keeping with both local and regional studies and surveys, most notably the SANHANES study [56], the estimate is lower than other sub-Saharan countries where overall prevalence (57%) is even higher [57–59]. However, we are able to report this result with greater confidence given our quality assessment of the studies using the GRADE tool, which allowed us to evaluate our result as ***important*** (Additional file 57). While anaemia plays a major role in contributing to global disease burden, the brunt is felt in lower income to middle-income countries (LMIC), particularly Africa and Asia, where the prevalence of anaemia in pregnant women can range between 46.3 and 60%. Our study showed that the prevalence in SA is in the range of 20 to 40% and so the country appears to be relatively less affected as compared to much of Africa. This is due in part to the slightly improved socio-economic status of South Africa as compared to other African countries. However, when compared to high-income settings such as Europe and the Americas, where the prevalence of anaemia is approximately 25% [59], this effect of economic disparity on health outcomes is made even clearer.

### Outcome: severity of anaemia

Nine studies reported on the severity or the grade of anaemia—either mild, moderate or severe, although they were not consistent in the manner of reporting. Although the heterogeneity was high (*I*^2^ 100%), the prevalence per grade (mild 59%, moderate 38% and severe 3%) were in keeping with the general findings in the literature.

The majority of patients presented with mild anaemia that progressed during the pregnancy [15, 18–20, 48, 49, 51, 60], with the apparent cause being attributed to iron deficiency or infection. While a bone marrow aspirate is the most definitive diagnosis of iron deficiency anaemia [61], this test is invasive and expensive. Hb concentration is used as a proxy measurement instead, given its low cost and ease of use. Without confirmatory iron studies, iron deficiency is often assumed to be the cause. While such deficiency may be attributed to the development of the placenta and growing demands of the foetus, it may also be due to the modulation of the maternal immune system [61] due to infective or inflammatory conditions [51, 62, 63].

Study-level subgroup analyses based on year of publication, race, quality of study and HIV indicate that the heterogeneity was pervasive.

The pooled prevalence of anaemia of 26% (*I*^2^ 96%) calculated for studies between 1970 and 1994 was lower than that of studies from 2003 to date (33%, *I*^2^ 98%), while the lowest prevalence was observed between 1995 and 2002 (7%). However, there were only two studies in this intermediate grouping, and although the heterogeneity was low (*I*^2^ 22%), we discounted any observed effect due to the year of study publication. This increase in prevalence could be indicative of an absolute increase because of the rising epidemics of HIV and Tb during this period, or worsening disparities in socio-economic status. It could also be due to better evaluation of anaemia using more sensitive tests or an increase in access to healthcare services post advent of democracy.

With regard to race, a pooled prevalence of 28% was noted in 13 studies that either included all race groups or did not differentiate the study population into race groups (*I*^2^ 100%), as compared to the prevalence of 33% for Black women, 17% for Coloured and White women (9 and 3 studies) and 49% for Indian women (Additional file 1). Again, significant heterogeneity was demonstrated amongst the first two groups (*I*^2^ 99% and 84%), with a moderate level of heterogeneity in the study that focussed on Indian women (*I*^2^ 48%). However, as with the subgroup analysis by year, this latter group only consisted of two studies, and the absolute effect was not further considered. Earlier studies which attempted to assign a racial association for anaemia based on diet or cooking practices were disproved, given that anaemia is more prevalent in Black pregnant women (33%). These results highlight the association between health status and the social determinants of health, in particular the impact that inequities in socio-economic status, access to safe water, adequate sanitation good nutrition and health care of an acceptable standard, or ethnic differences can have on a population [64]. The present estimate may be more reflective of the effects of lack of sanitation and safe water that could predispose to infective causes of anaemia, rather than ethnic differences alone.

### Outcome: risk factors for anaemia—HIV, TB and other co-morbidities

Risk factors for anaemia included HIV, TB, and parasitic infections in particular, and then any other comorbidities in general. Subgroup analyses of prevalence of anaemia in pregnant women in those papers that reported on HIV (*n* = 13) were compared to the 13 that did not. The pooled prevalence of anaemia (28%) in the latter group was understandably lower, than the 38% in the former, since this group included papers published prior to the first reported cases of HIV between 1982 and 1985. Heterogeneity was high in both groups—*I*^2^ 99% and 98% respectively.

Risk factors for anaemia other than iron deficiency should be considered, including the social determinants of health which predispose to common infections such as HIV and AIDS, TB and parasitic infestations, as well as obesity [3]. In the studies conducted in HIV positive cohorts, a positive association between anaemia and the condition was seen [5, 18–20, 31, 18, 34, 35, 37, 41, 44, 47, 51]. Globally, there is an increase in the prevalence of anaemia in HIV-positive patients and those with AIDS, possibly due to increased blood loss from concomitant neoplasia or gastrointestinal lesions, or due to decreased red blood cell (RBC) production, RBC destruction or inefficient RBC production. These findings were supported by this review. The Kesho Bora Study Group [47] showed that South African pregnant women who were HIV positive had, like their African counterparts, an increased prevalence of anaemia related to socio-economic status, advanced disease, and prolonged duration of disease, although this risk was decreased compared to Kenya, given that malaria is not endemic in this setting. Nandlal [49] showed a similar finding in relation to lower CD4 counts in a cohort of pregnant South African HIV women, as did Tunkyi and Moodley [51], and further demonstrated that the severity increased with gestational age. These factors had a significant negative impact on both maternal and foetal outcomes.

South Africa has a high burden of TB infection, with an estimate of 301,000 active cases in 2018 [65]. The association of anaemia in patients with TB [66, 67]: we found only one study that reported on TB as a mediator of anaemia status, with a high prevalence of 70% in the pregnant population [61]. This is a considerable deficit in the body of knowledge, especially since South Africa is a LMIC and lower socio-economic status is associated with increased TB risk, as also shown in similar settings [68].

While the earlier studies in this review [38, 53–55] advocate routine supplementation, later work from Hoque [36], Tunkyi and Moodley [20] and Symington et al. [5] all argue that other risk factors need to be ascertained given the high infective burden of schistosomiasis, helminths and other parasitic infections. These infections lead to the sequestration of circulating iron to protect the host from fulminant infection, but may result in anaemia [15]. Indeed, Hoque [69] demonstrated a 12-fold increase in the risk of anaemia in the presence of schistosomiasis infection.

### Outcome: hypertensive disorders of pregnancy

Anaemia has been shown to be associated with HDP, adversely affecting both maternal and perinatal outcomes [4, 14]. The greater the severity and duration of anaemia, the higher the risk for HDP as well as resultant maternal and foetal morbidity and mortality [20]. While only four studies reported on hypertension and anaemia, none showed a statistically increased risk for HDP. However, Nandlal [51] and Tunkyi and Moodley [47] showed that HIV-positive women—already at risk for anaemia associated with the infection—were more likely to develop HDP, as well as a nonsignificant increase in abruptio placentae when on antiretrovirals needed to treat the condition. The severity of anaemia increased with the duration of anti-retroviral treatment [70]. Thus, it is important to manage anaemia both in the non-pregnant population, as well as make an early comprehensive diagnosis of anaemia and manage appropriately in early pregnancy so as to limit the effects and unwanted sequelae to both mother and baby.

### Outcome: low birth weight

Of the nine studies that reported on birth outcomes, eight were also associated with HIV-positive mothers [5, 18, 19, 34, 4, 35, 41, 44, 48]. Low birth weight and preterm labour were the most common adverse events, but it was difficult to ascertain whether this was directly due to the anaemia, the infection or both, as well as in which direction the association might be. Our finding is thus in keeping with the general body of evidence, which at present remains inconclusive [51].

### Outcome: anaemia and supplementation

Although a Cochrane review [71] reported that there was no reduction in risk following prenatal supplementation, Haider et al. [14] report significant evidence to the contrary. They go on to postulate a biological model for plausible support for the theory, both in terms of maternal gut absorption of dietary iron in the face of depleted iron stores as well as placental transfer to the foetus, showing a linear dose response between dose and resultant birth weight. Our findings would seem to indicate that it is anaemia per se, and not necessarily iron deficiency anaemia that has negative impacts on birth outcomes. However, in the presence of other comorbidities, it is difficult to ascertain cause and effect, as well as impact.

Anaemia in pregnancy can result in immediate, medium and long-term adverse effects on maternal and foetal wellbeing and obstetric outcomes [34]. Multisectoral interventions implemented at a sociodemographic level can result in a reduction in the burden of disease of anaemia and its long-term and widespread sequelae in the population as a whole. Health promotion strategies together with routine screening and mass treatment of populations for common infections need to be considered interventions to be implemented at the primary health care (PHC) level, particularly as part of the antenatal package of care.

## Limitations

This is the first study to systematically review the available literature on the prevalence of anaemia in South African pregnant women; nonetheless, several limitations exist. Firstly, there is a variation in the period of gestation at which anaemia was assessed (first antenatal visit or subsequently) and the type of diagnostic test used. Although anaemia progresses with pregnancy, many in this population seek antenatal care late in the pregnancy, and the overall severity reported might actually be underestimated due to the physiological changes of pregnancy itself.

There was a paucity of data reporting on prevalence of anaemia and direct maternal and foetal outcomes. The cross-sectional study design of the majority of studies selected meant causal associations with maternal and foetal outcomes could not be ascertained. Demographic data of interest was not available for most study populations and as such could not be reported on. This would have contributed to the heterogeneity amongst studies in the subgroup analyses.

Generalisability may be limited, since all studies were conducted in women attending public health sector settings, and not the private sector. Anaemia may be due to other causes in this more affluent population, which warrants further investigation. Furthermore, most studies were set in two of the 11 provinces namely KwaZulu-Natal and Gauteng; therefore, the pooled prevalence may be skewed towards these provinces.

## Conclusions and recommendations

From the pooled prevalence reported in this study, we estimate anaemia in the pregnant population to be 31% (95% CI, 23–39%). This can be extrapolated to the general population. While the prevalence of anaemia remains high, risk factors are varied. Iron deficiency is still common but the presence of comorbidities in South Africa also contributes to anaemia and should not be ignored. Pregnant women should be assessed for causes of anaemia other than iron deficiency. Further longitudinal studies at the local level need to be undertaken to more carefully unpack these associations and should include data on appropriate tests used to determine anaemia and establish iron status, supplementation regimens and compliance by the patient and the period of gestation as well as evaluate maternal comorbidities and adverse birth outcomes.

Evidence exists that there may be other reasons for anaemia, including infection and infestation. Simple screening for parasites should be done at the PHC level, incorporated into antenatal care. Thus, guided interventions can be implemented so as to effect the best outcome, rather than ubiquitous supplementation with iron.

## Supplementary Information

**Additional file 1.** Sub group analysis by province.

**Additional file 2.** Search strategy.

**Additional file 3.** Modification of the Hoy Tool* for risk of bias in prevalence studies.

**Additional file 4.** DOI plot and LFK ratio for publication bias and asymmetry of overall pooled prevalence.

**Additional file 5.** Characteristic of studies included in this meta-analysis.

**Additional file 6.**

## Data Availability

All data generated or analysed during this study is included in the systematic review article and will also be available upon request

## Abbreviations

ANC: Ante natal care/clinic
GRADE: Grading of Recommendations Assessment, Development and Evaluation
RCT: Randomised control trial
Hb: Haemoglobin
HDP: Hypertensive disorders of pregnancy
LBW: Low birth weight
LMIC: Low- to middle-income country
MCV: Mean cell volume
MeSH: Medical Subject Headings
TB: Mycobacterium tuberculosis
PRISMA: Preferred Reporting Items for Systematic Reviews and Meta-analysis
PIH: Pregnancy-induced hypertension/gestational hypertension
PROSPERO: Prospective Register of Systematic Reviews
SARS-CoV-2: Severe acute respiratory syndrome coronavirus 2
SA: South Africa/n
SDGs: Sustainable development goals
WHO: World Health Organisation
