# Supplementary material for: The prevalence, risk factors and outcomes of anaemia in South African pregnant women: a systematic review and meta-analysis
Source: Syst Rev. 2022 Jan 25;11:16. doi: 10.1186/s13643-022-01884-w (PMC8789334; doi:10.1186/s13643-022-01884-w)
Supplement: Supplementary file 1 — Additional file 1. Sub group analysis by province. [file 13643_2022_1884_MOESM1_ESM.docx]

Additional File 1. Sub group analysis by province
