# Supplementary material for: The prevalence, risk factors and outcomes of anaemia in South African pregnant women: a systematic review and meta-analysis
Source: Syst Rev. 2022 Jan 25;11:16. doi: 10.1186/s13643-022-01884-w (PMC8789334; doi:10.1186/s13643-022-01884-w)
Supplement: Supplementary file 2 — Additional file 2. Search strategy. [file 13643_2022_1884_MOESM2_ESM.docx]

Additional file 2. Search strategy

| Database used | Search terms | Articles found |
| --- | --- | --- |
| PubMed | (((((((((“anaemia”) OR “anemia”) OR “haemoglobin”)  OR “hemoglobin”) OR “iron deficiency”) OR  “haematocrit”) OR “hematocrit”)) AND  (((((((((((“pregnancy”) OR “pregnant”) OR  “maternal”) OR “mother”) OR “gravid”) OR  “maternal exposure”) OR “obstetric”) OR  “antenatal”) OR “antepartum”) OR “gestation”) OR  “gestational”)) AND (((“south africa”) OR “southern  africa”) OR “south african” OR “Sub-Saharan”) | 486 |
