# Supplementary material for: The prevalence, risk factors and outcomes of anaemia in South African pregnant women: a systematic review and meta-analysis
Source: Syst Rev. 2022 Jan 25;11:16. doi: 10.1186/s13643-022-01884-w (PMC8789334; doi:10.1186/s13643-022-01884-w)
Supplement: Supplementary file 3 — Additional file 3. Modification of the Hoy Tool* for risk of bias in prevalence studies. [file 13643_2022_1884_MOESM3_ESM.docx]

| 1. Was the study's target population a close representation of the national population in relation to relevant variables? | - Yes (LOW RISK) - No (HIGH RISK |
| --- | --- |
| 1. Was the sampling frame a true and close representation of the target population? | - Yes (LOW RISK) - No (HIGH RISK |
| 1. An appropriate sample size was used? | - 200 :Yes (Low risk) - < 200: No (High risk |
| 1. Was the likelihood of non-response bias minimal? | - Yes (LOW RISK) - No (HIGH RISK) |
| 1. Did the study evaluate limitations to extrapolation of prevalence to population? | - Yes (Low Risk) - No (High Risk) |
| 1. Were data collected directly from the subjects (as opposed to a proxy)? | - Yes (Low Risk) - No (High Risk) |
| 1. Was an acceptable case definition used in the study? | - Yes (Low Risk)   No (High Risk) |
| 1. Was the study instrument that measured the parameter of interest (e.g. prevalence of anaemia) shown to have reliability and validity (if necessary)? | - Yes (Low Risk)   No (High Risk) |
| 1. Was the same mode of data collection used for all subjects? | - Yes (Low Risk)   No (High Risk) |
| 1. Were the numerator(s) and denominator(s) for the parameter of interest appropriate? | - Yes (Low Risk)   No (High Risk) |
| 1. Overall assessment of bias from analysis | Quality score |

Additional file 3. Modification of the Hoy Tool* for risk of bias in prevalence studies

*Adapted from Hoy D, Brooks P, Woolf A, Blyth F, March L, Bain C, et al. Assessing risk of bias in prevalence studies: modification of an existing tool and evidence of interrater agreement. J Clin Epidemiol. 2012;65:934–9.
