# Supplementary material for: The prevalence, risk factors and outcomes of anaemia in South African pregnant women: a systematic review and meta-analysis
Source: Syst Rev. 2022 Jan 25;11:16. doi: 10.1186/s13643-022-01884-w (PMC8789334; doi:10.1186/s13643-022-01884-w)
Supplement: Supplementary file 5 — Additional file 5. Characteristic of studies included in this meta-analysis. [file 13643_2022_1884_MOESM5_ESM.docx]

| **Additional file 5: Characteristic of studies included in this meta-analysis.** | | | | | | | | | | |
| --- | --- | --- | --- | --- | --- | --- | --- | --- | --- | --- |
| Author, Year | N | Prevalence of Anaemia (%) | Causes | Tests employed | How was anaemia established? | Province | Race | Study Design | Relevant findings | Conclusion |
| Akinsooto 2020 | 150 | 48.0 | Iron deficiency | Lab | Hb | KZN | Did not report | C/S | Soluble transferrin receptor is highly sensitive and specific in detection of iron deficiency anaemia and is independent of inflammation or infection | Soluble transferrin receptor may be a useful diagnostic tool to confirm iron deficiency anaemia |
| Becker 1970 | 128 | 13.3 | Iron deficiency | Oxyhaemoglobin method | Transferrin | Gauteng | Black | C/S | Black females are able to meet the iron demand of pregnancy from iron stores in the liver and bone marrow but this may not be always the case. | At term, no significant difference was noted in e median Hb concentration between the treated and untreated groups. However, all indices lower in untreated group |
| Bloch 2020 | 135 | 25.0 | Not reported, Indications for blood transfusion, OH, termination | Clinical record review | Hb | KZN, Gauteng | Black, Coloured | C/S | Unaddressed antenatal anaemia is a significant risk factor for later maternal transfusion. These patients areless likely to tolerate physiological blood loss that accompanies delivery. Anaemia is also a risk factor for poor infant outcomes including cognitive impairment. Anaemia more common in HIV positive women | Opportunities for practice improvements:  an intervention to screen antenatal women more aggressively, with administration of haematinics (e.g., iron, folate) if so indicated |
| Bopape 2008 | 123 | 36.0 | Iron and Folate deficiency, Megaloblastic | Coulter STKS | B12 | Limpopo | Black | C/S | The prevalence of anaemia was 36%, which is higher than the prevalence of 26% that was reported among pregnant adult women residing in Bloemfontein and the 20.5% prevalence among pregnant adult women in Soweto. | The prevalence of anaemia was high in the study population, with deficits in intake of iron, folate and vitamin C in diets of study population reported. |
| Dommisse 1969 | 45350 | 10.1 | Iron deficiency, Megaloblastic | Lab | Hb | WC | All | C/S | Iron deficiency anaemia most common among pregnant women with severe anaemia with megaloblastic erythropoiesis in 3% of severe anaemia | As iron deficiency is common, haematinics are recommended in all pregnant women but folic acid should be reserved for selected cases |
| Donald 2019 | 116 | 17.0 | Classified as ID | Haemoglobin | Hb | WC | Did not report | L/inal | Key maternal and child physical health risk factors for lower developmental scores or higher odds of delay included maternal anaemia, prematurity, maternal HIV, alcohol or tobacco use during pregnancy. Maternal anaemia in pregnancy was associated with lower cognitive scores, receptive language scores and expressive language scores, with a greater negative effect in boys than for girls | Maternal anaemia, one of the pregnancy-related risk factors for which there is robust evidence for long-term developmental impact, was associated with poor developmental outcomes, in boys more than girls. . |
| Govender 2020 | 314 | 31.8 | Not reported | Retrospective chart review | Not reported | KZN | Did not report | C/S | Late ANC booking and reduced ANC visits were common for adolescent pregnancies. Under-utilisation of ANC (i.e. fewer than 4 visits) was significantly associated with lower gestational age low birthweight, low Apgar scores as well as maternal anaemia | Adolescent pregnancy in this research setting was associated with a risk of late booking and reduced ANC visits, which could lead to adverse maternal and birth outcomes. |
| Hoque 2007 | 1214 | 55.7 | Not reported, Iron deficiency most likely cause | Coulter counter | Hb | KZN | Black | C/S | In terms of the South African National Department of Health definition of anaemia in pregnancy, (haemoglobin < 10 gm/dL), 30.1% of the attendees were anaemic. According to the World Health Organisation (WHO) criteria (Hb < 11 gm/dL), the prevalence of anaemia was 57.3%. The difference in the prevalence of anaemia on the basis of the two different criteria was significant (p = 0.000). This finding was comparable to studies conducted in other Sub-Saharan African countries. | The prevalence of anaemia in pregnancy in this community is high. The prevalence of anaemia varied greatly when the WHO definition was used. The pregnant women of this community attend the healthcare facility very late for their booking visit, in spite of free maternity services at public health facilities. There is an urgent need for health education and health promotion programmes in this population for early booking for antenatal care. Further investigations are needed to establish the risk factors for, causes of and preventive interventions for anaemia in pregnancy. |
| Kerkhoff 2014 | 20 | 70.0 | Not reported. ? Ineffective | Lab | Hb | WC | Did not report | C/S | High prevalence of anaemia among treatment-naive patients enrolling to start ART. A substantial proportion of those with anaemia had underlying TB, which was a strong independent risk factor for anaemia. The prevalence of TB among those with moderate or severe anaemia was so high and their clinical outcomes were so poor that it suggests the need for routine microbiological investigations for TB in this patient group. | A very high prevalence of undiagnosed TB was found in patients with moderate or severe anaemia. Such patients should be prioritized for routine microbiological investigation using rapid diagnostic assays. |
| Kesho Bora 2013 | 284 | 57.8 | Not reported. ?HIV | Did not report | Hb | Did not report | Did not report | RCT | Among HIV-positive observational cohort women who were free of severe anaemia at baseline, only socioeconomic status category was independently associated with severe anaemia risk. These included local setting (country), more advanced HIV disease, prolonged breastfeeding and duration of ARV. The observed reduced anaemia risk in South African and converse elevated risk in Kenya compared to Burkina Faso, is likely explained by the fact that little or no malaria exists in the study sites in South Africa while malaria-associated anaemia is common in the Kenyan site | Similar severe anaemia risk exists when comparing the short and longer ZDV containing regimens. This should reassure policy makers who are increasingly recommending longer ARV regimens for PMTCT in low- and middle-income countries. The importance of key independent predictors of anaemia confirmed, showing the groups that require closer monitoring. ARV use for as little as one month may be beneficial to reduce severe anaemia risk among pregnant (and postpartum) women and may provide long term protection following cessation of prophylaxis. |
| Lamparelli 1988 | 224 | 11.6 | Iron deficiency, Genetic, 3 with heterogenous B thalassaemia | Multiple instruments for FBC, iron and ferritin | Hb, MCV, Ferritin, Iron studies | Gauteng | Indian | C/S | While supplementation of iron will help during pregnancy, effort needs to be made to improve overall iron intake prior to pregnancy in the female Indian population via fortification. The mean haemoglobin concentration and packed cell volume showed a significant decline with progression of gestation. The total iron-binding capacity showed a highly significant increase as pregnancy advanced (P < 0,01). The geometric mean serum ferritin concentrations in all three trimesters were below 12 µg/1. Using this value as an arbitrary cut-off, 43,3% of women in the first, 48,6% in the second and 80% in the third trimester of pregnancy had depleted iron stores. The percentage saturation and iron stores in the third trimester were significantly lower than in the first (P < 0,05). | Methods of iron fortification need to be explored that will help improve iron stores in this population without it influencing other populations who are at risk of iron overload. By the third trimester, 20% of the study population had iron deficiency anaemia. underlining the need for routine iron supplementation of pregnant Indian women in the Johannesburg area. |
| Lamparelli 1988b | 100 | 8.0 | Iron deficiency, Megaloblastic | Coulter Model S | Hb, MCV, Ferritin, Iron studies | Gauteng | Coloured | C/S | Pregnant coloured women have depleted iron stores early in pregnancy. reflective of the iron status of non-pregnant population leading to iron deficit later in pregnancy. Haemoglobin concentrations< 11 g/dl were present in 18,9% of women in the third trimester of pregnancy, while 64% had a saturation of transferrin value of< 16% and 68% a serum ferritin level < 12 µg/1. Calculations suggested that mean iron stores in the first trimester were 228 mg, with 37,5% of women having absent stores. Comparable figures in the second and third trimesters were 74·mg and -92 mg respectively. The fact that many were iron deficient in the first trimester indicates a high frequency of iron deficiency in non-pregnant women in this population group. Although 20,8% of the women had red cell folate values below the normal range for non- pregnant subjects, folate deficiency did not appear to be a significant problem. Vitamin B12 deficiency was very uncommon. | Frank and overt iron deficiency was prevalent in 10% of population group and megaloblastic anaemia indicating folate deficiency may have been masked by microcytosis due to iron deficiency. Routine supplementation in required in this population group. There is a relatively high prevalence of iron deficiency in coloured women in Johannesburg, a group which has been little studied in the past. By WHO criteria, there was a 4% prevalence of significant anaemia in early pregnancy which rose to approxi­mately 19% in late pregnancy. Routine iron supplementation is clearly indicated in pregnant women of this population group. |
| Levy 2018 | 102 | 50.0 | Iron deficiency | Sysmex X 9000 | Hb, Ferritin, Iron studies | Gauteng | Black | C/S | Using Hb alone to determine iron deficiency anaemia may not be appropriate as subclinical iron deficiency may be missed. | Using flow cytometric determination of reticulocytes and hypoHb% is comparable to more expensive gold standard techniques that limits its use for determination of iron deficiency which may be more prevalent in the population than is diagnosed by WHO classification using Hb or ferritin. |
| Macaulay 2018 | 1804 | 28.0 | Not reported | Venous blood for Hb | Hb | Gauteng | Black | C/S | Women diagnosed with GDM had significantly higher weights and body mass indexes (BMIs), were significantly older, of higher household socioeconomic status, more likely to report a family history of diabetes, and more likely to be diagnosed with anaemia than women without GDM. | A diagnosis of GDM increases the risk of both mother and child developing Type 2 diabetes which causes further health complications, decreases longevity, and burdens a country’s healthcare system. Therefore, a GDM prevalence of 9.1% is concerning and warrants further discussion around current GDM screening policies. |
| Mathee 2014 | 307 | 16.9 | Not reported | Did not report | Hb | Gauteng | Black | C/S | Geophagic practice is common in South Africa (22.8%). May remain undisclosed due to fear of censure, or openly admitted. Significantly associated with increased lead consumption | Geophagia is practised by a considerable proportion of pregnant women in Johannesburg, especially migrant women. Greater vigilance in respect of pica, especially geophagia, may be needed as part of antenatal care programmes to avoid potentially detrimental health effects of the practice. |
| Mayet 1985 | 263 | 16.0 | Iron deficiency, Megaloblastic | Venous blood using Coulter counter | Hb, Ferritin, Iron studies, Transferrin saturation | KZN | Black, Indian | C/S | Anaemia was present in 13,2% of Indian women and in 18,8% of blacks in the first trimester of pregnancy. There was a progressive increase in the prevalence of anaemia as pregnancy advanced, particularly in the case of the Indians, in whom the highest prevalence (47%) was noted in the third trimester, while 28,6% of the black women were found to be anaemic in late pregnancy. Anaemia was not only commoner but also more severe in late pregnancy. | Anaemia was common in this group and the prevalence increased progressively as pregnancy advanced. Iron deficiency was by far the commonest type of deficiency observed. While folate levels were low in a fair proportion of subjects, evidence of coexistent iron deficiency was found in all. It is therefore not clear whether or not a primary nutritional deficiency of folic acid contributed towards the production of anaemia. It could be recommended on the basis of this study that iron tablets should be given to all pregnant women in both ethnic groups. A case could possibly be made for the routine administration of folic acid to black women, among whom low serum folate levels tended to occur in the absence of iron deficiency. |
| Mkhize 2019 | 200 | 44.5 | Iron deficiency, Not reported | Lab | Hb, Ferritin | KZN | Black | C/S | Non-adherence to oral supplements is high but supplementation was not associated with LBW. Anaemia was more prevalent in HIV positive group | Non-adherence to iron and folic acid supplementation in pregnant women was higher in HIV-positive than HIV-negative women. Nausea was the commonest side effect across all trimesters. Patient education and pill counting devices should be used. |
| Nandlall 2014 | 408 | 64.2 | Not reported | FBC | Hb | KZN | Black | L/inal | The mean Hb concentration was 10.6 g/dL at baseline and 262/408 (64.2%) women were diagnosed with anaemia (Hb11 g/dL) in pregnancy, 48/146 (32.9%) subsequently developed anaemia intrapartum or postpartum and 89/310 (28.7%) of all cases of anaemia remained unresolved by 2 weeks post-delivery. CD4 count and gravidity were significant risk factors for anaemia in pregnancy. | Anaemia was most common among women in the advanced stage of HIV infection (CD4,200 cells/mm3). There was no evidence of an association between ZDV or triple ARVs and anaemia. |
| Notelovitz 1972 | 171 | 16.0 | Iron deficiency | Transferrin saturation | Hb, Transferrin saturation | KZN | White, Coloured | C/S | The results indicate that the haemoglobin is a poor index of iron nutrition as measured by total saturation. Many patients had normal haemoglobin levels with depleted iron stores. This was true also for other haematological values studied. | Anaemia, and in particular iron deficiency anaemia, was more common among Coloureds. |
| Ross 1981 | 1051 | 35.2 | Iron deficiency | Coulter counter | Hb, Ferritin | KZN | Black | C/S | The mean haemoglobin value at booking 11,9 g/dl. Parity did not influence values at booking and no one parity group was at greater risk of a decline in values. Older women tended to have lower values than younger ones. Older patients were not more likely to suffer a drop in values than younger women. There was a highly significant (P= 0,0002) direct correlation between declining haemoglobin values and number of antenatal clinic attendances, a reflection of the fact that patients with the largest number of attendances booked earlier in pregnancy and physiological haemodilution was therefore more apparent in this group. | While up to 10% of pregnant women were found to have haemoglobin values of less than 10 g/dl, less than half these had more than 50% microcytic red cells. Serum ferritin levels were pathologically low in only 5% of patients and indicate that in most cases iron stores were adequate. The lack of correlation between serum ferritin levels and haemoglobin values would also indicate that low haemoglobin values due to iron deficiency were not a common problem. Failure of haemoglobin values to respond to the use of prophylactic iron could also be taken as indicative of the absence of a significant degree of iron deficiency. Unless iron deficiency anaemia in pregnancy has been shown to be a common problem in a community or a section of a community it is surely unjustifiable to attempt routine prophylaxis. Amore satisfactory policy would be to identify patients who have low haemoglobin concentrations, find out in which individuals this is due to pathological change, and treat the cause |
| Symington 2019 | 250 | 29.0 | Iron deficiency | Hb, ferritin and sFtr | Hb, Ferritin, Iron studies | Gauteng | All | C/S | The prevalence of anaemia, iron deficiency (ID) and iron deficiency erythropoiesis (IDE)increased despite iron supplementation forming part of routine antenatal care. ID and anaemia at mid-pregnancy, as well as IDE at late-pregnancy were associated with higher birth weight. In contrast, women with IDE at mid-pregnancy had a 3.6 times higher risk of giving birth prematurely and women with a lower haemoglobin at early pregnancy gave birth significantly earlier than those in the highest haemoglobin quartile. | There was an increase in ID, IDE and ID anaemia with pregnancy progression despite routine iron supplementation in an urban South African setting. An inverse association between maternal iron status and birth weight was observed, while IDE at mid-pregnancy increased the risk for premature birth. These results add to the raising concern on the consequences of iron supplementation in iron-replete pregnant women. Nonetheless, there is no question that ID and anaemia should be prevented in pregnancy. However, the challenge remains on how to do so safely in a public health setting. Considering that South Africa has a well-implemented food fortification programme, high prevalence of inflammation, possible influence of antenatal calcium supplements on iron absorption, as well as the known risks associated with both low and high iron exposure, it is recommended that the current antenatal supplementation regime in South Africa be revisited. |
| Tunkyi 2015 | 2000 | 42.7 | Not reported | Lab | Hb | KZN | Did not report | C/S | The prevalence of anaemia in pregnancy at the first antenatal visit in the study cohort of 2 000 pregnant women was 42.7%, HIV infection has been reported to be associated with anaemia, either independently or due to antiretroviral medications such as zidovudine. At 32–34 weeks of gestation, 403 (28.1%) of the 1433 pregnant women had documented Hb levels of Hb, <11 g/dl. One hundred eighty (22.8%) of the 789 who were non-anaemic at first antenatal visit developed anaemia at 32–34 weeks, while 609 (77.2%) remained normal. A higher prevalence of anaemia 42.7% was observed at the booking and decreased to 28.1% at 32 weeks. | The prevalence of anaemia at the first antenatal visit was high and a major health issue. There is a need to strengthen the healthcare system to ensure a definitive diagnosis so that appropriate counselling and treatment can be provided in early pregnancy. The prevalence of anaemia decreased from 42.7% (booking) to 28.1% (3234weeks). Normocytic normochromic anaemia was the commonest type of anaemia. Attention needs to be focused on detailed investigations to establish the exact cause of anaemia. |
| Tunkyi 2017 | 2000 | 42.7 | Not reported | Did not report | FBC | KZN | Did not report | C/S | The prevalence of anaemia in HIV-infected women was 64.6%. The prevalence of anaemia in those patients who tested HIV-seropositive for the first time was 34.5% and may be a more accurate reflection of their Hb concentration levels. 41% of HIV-positive pregnant women with anaemia were on ARV treatment, compared to 92% of HIV-infected. Most of the types of anaemia were normocytic normochromic in type suggesting that iron deficiency is not a major factor in the aetiology of the anaemia and women with anaemia who were not on ARV treatment. The prevalence of anaemia was 42.7% at the first antenatal visit. Thirty-five percent had mild anaemia and 68.9% had normocytic normochromic anaemia. The prevalence of anaemia in HIV infected women was higher than that in the noninfected group and 47.2% of the study population (n = 2000) was HIV infected. At the 32–34 weeks visit, haemoglobin (Hb) levels were available for 1433/2000 (71.7%) of the participants. The prevalence of anaemia was 28.1% (n = 403/1433); 19.3% had mild anaemia and 65.3% had normocytic normochromic anaemia. There was a significant difference in Hb levels between that of the first visit and that at 32–34 weeks (42.7% vs. 28.1%; p ¼ .001; 95% CI: 0.11–0.18). There were significant differences in prematurity, birth weight and hypertensive disorders of pregnancy between the anaemic and non-anaemic groups. | Anaemia is a common finding in pregnant women diagnosed with HIV with high perinatal and maternal morbidity rates. Assessment of anaemia at the first antenatal visit is therefore essential as it affords one the opportunity to establish the exact cause of low Hb concentration levels, and institute interventions to prevent complications. It must be recognised, however, that anaemia is not a diagnosis and must prompt detailed investigations for the underlying cause in particular chronic infections. Severe anaemia in pregnancy is associated with poor pregnancy outcomes and the exact cause or association is yet to be elucidated. |
| Tunkyi 2018 | 2000 | 42.7 | Iron deficiency, normocytic normochromic | Lab | Hb, FBC | KZN | Did not report | L/inal | Of the 2 000 recruited, 854 (42.7%) were anaemic, 943 (47.2%) were infected with HIV, and 609 (64.6%) HIV-infected women had anaemia. There was a significant difference in the prevalence of anaemia in HIV-infected patients on antiretroviral (ARV) treatment compared to untreated patients (41.4% vs 92.1%;p < 0.0001). Mild grades of anaemia were common in HIV-infected patients on ARVs, while moderate to severe grades were most common in patients who were not on ARVs. Besides birthweight and hypertensive disorders of pregnancy, there was no significant difference in neonatal and maternal outcomes irrespective of duration of ARV treatment | There was a high prevalence of anaemia among HIV-infected, untreated pregnant women. Assessment of anaemia at the first antenatal visit is, therefore, essential. |
| van Bogaert 2006 | 3214 | 19.7 | Not reported | Did not report | Hb | Limpopo | Black | C/S | There were only 112 women with recorded evidence of iron and folate supplementation. The overall prevalence of anaemia was 19.7%. Multigravidas with SVD had a 22.5% prevalence of booking Hb <10 vs 14.2% in primgravidas. The prevalence of severe anaemia was similar for all subgroups. No significant correlation between the booking Hb and pregnancy outcome in terms of birth weight or mode of delivery. However, booking visit anaemia could predispose to caesarean delivery. Documented iron and folate supplementation did not appear to result in different pregnancy outcomes. | Anaemia at the booking visit may predispose to caesarean delivery. Documented iron and folate supplementation did not appear to result in different pregnancy outcomes |
| Weyers 2016 | 104 | 33.0 | Iron deficiency, ?other causes |  | Hb, Ferritin, MCHC RetHE | Free State | Did not report | C/S | Thirty-two percent of study participants were HIV-positive, reflecting the prevalence of HIV infection in the Free State Province of South Africa A large number of anaemic participants (47.1%) fell in Quadrant 1 of the diagnostic plot. There were probably other causes for anaemia in these patients (such as gestational anaemia or concomitant nutritional deficiencies). The possibility of HIV-related myelodysplasia should also be considered. 12.6% of participants already had iron-deficient erythropoiesis at the first antenatal visit. | Patients presenting for their first visit when already in the third trimester of pregnancy are at an especially high risk for iron-deficient erythropoiesis. Being HIV-positive did not affect the iron status of the pregnant patient population. In anaemic pregnant women, causes other than iron deficiency should be considered and serum ferritin is not a reliable indicator of iron stores. Routine laboratory tests have limitations in diagnosing iron-deficient states in pregnancy, but when using newer diagnostic tests (RET-He and sTfR assay) in the Thomas plot, it can greatly assist with the iron status assessment in complicated cases. |
| Wilkinson 1997 | 300 | 5.0 | Not reported | Haemoglobinometer | Hb | KZN | Did not report | C/S | CuSO4 test can be used to identify risk of anaemia which can be confirmed by haemoglobinometer which is cost effective and efficient | This study shows that the combined use of copper sulphate solution supplemented with a haemoglobinometer, is a feasible, accurate and cost-effective way of screening for and diagnosing anaemia in pregnancy on site in primary care clinics. With this strategy, most women without anaemia will be correctly screened out by the copper sulphate solution, and those who are possibly anaemic will have their true haemoglobin concentration measured. This can then be acted upon, according to local protocols, with only severe cases being referred for further examination and investigation. |
