# Supplementary material for: The prevalence, risk factors and outcomes of anaemia in South African pregnant women: a systematic review and meta-analysis
Source: Syst Rev. 2022 Jan 25;11:16. doi: 10.1186/s13643-022-01884-w (PMC8789334; doi:10.1186/s13643-022-01884-w)
Supplement: Supplementary file 6 — Additional file 6. [file 13643_2022_1884_MOESM6_ESM.docx]

**Additional file 6**

**Author(s)**: Dorsamy, Bagwandeen, Moodley

**Question**: The Prevalence, Risk factors and Outcomes of Anaemia in South African Pregnant Women: A Systematic Review and Meta-analysis

**Setting**: South Africa

**Bibliography**:

| **Certainty assessment** | | | | | | | **№ of patients** | | **Effect** | | **Certainty** | **Importance** |
| --- | --- | --- | --- | --- | --- | --- | --- | --- | --- | --- | --- | --- |
| **№ of studies** | **Study design** | **Risk of bias** | **Inconsistency** | **Indirectness** | **Imprecision** | **Other considerations** | **anaemia** | **non-anaemia** | **Relative (95% CI)** | **Absolute (95% CI)** |  |  |
|  | | | | | | | | | | | | |
| 26 | observational studies | serious ^a^ | not serious | not serious | not serious |  | 13624/59235 (23.0%) | 45611/59235 (77.0%) | **OR 23.00** (0.11 to 0.38) | **217 more per 1,000** (from 501 fewer to 210 fewer) | - | IMPORTANT |

**CI:** Confidence interval; **OR:** Odds ratio

#### Explanations

a. Study design prone to bias
